# Resalinization Is Associated with Bacterial Alpha Diversity by Influencing Life-History Strategies, and Niche Properties in Coastal Saline Soils

**DOI:** 10.3390/biology15201816

**Published:** 2026-10-12

**Authors:** Xueya Liu, Honghao Cheng, Mengli Liu, Chong Wang

**Affiliations:** 1 College of Resources and Environmental Sciences, China Agricultural University, Yuanmingyuan West Road, Beijing 100193, China; 2 National Center of Technology Innovation for Comprehensive Utilization of Saline-Alkali Land, Dongying 257029, China

**Keywords:** coastal saline soils, abundant taxa, life-history strategy, microbial community assembly, niche properties

## Abstract

Soil salinization is a critical global limiting factor constraining agricultural productivity. Although soil salinity strongly influences microbial diversity and community composition, the underlying mechanisms regulating microbial diversity remain poorly understood. This work aimed to elucidate the impact of the resalinization process on bacterial alpha diversity in coastal saline soils. Soil samples were collected in the Yellow River Delta, Shandong, China, in March, May, and June, and soil salt content increased from 1.73 to 2.33 g kg^−1^ over this period. Our results indicate that soil salt content increased significantly across different periods (*p* < 0.01), exhibiting typical characteristics of soil salinization. Increased soil salinity was accompanied by a lower relative abundance of bacterial phyla with lower rrn copy numbers (low-rrn bacteria) and a higher relative abundance of bacterial phyla with higher rrn copy numbers (high-rrn bacteria) (*p* < 0.05). Moreover, high salinity was also associated with a decrease in niche breadth (*p* < 0.05) and an increase in niche overlap (*p* < 0.001) among high-rrn bacteria, whereas niche breadth increased and niche overlap decreased in low-rrn bacteria (*p* < 0.01). Consequently, the PLS-SEM (partial least squares structural equation modeling) showed that increasing soil salinity constrained bacterial resource utilization by regulating the niche properties of high- and low-rrn bacteria, thereby reshaping bacterial community structure and ultimately affecting bacterial α diversity. These findings provide a theoretical basis for improving soil microbial functioning by developing biologically based remediation strategies in coastal saline soils.

## Simple Summary

Coastal saline soils can accumulate salt during spring, which may affect soil health and bacterial communities. We investigated how seasonal salt accumulation was associated with changes in bacterial diversity and ecological characteristics in coastal saline soils in Shandong, China. We found that increasing soil salinity was associated with lower bacterial diversity, an increase in bacteria with higher growth potential, and a decline in bacteria with lower growth potential. Salt accumulation was also associated with stronger environmental selection and changes in how bacteria used available resources and space. These findings highlight the sensitivity of soil bacterial communities to seasonal salinity changes and provide useful information for managing and restoring coastal saline soils.

## 1. Introduction

Soil salinization in coastal areas has become increasingly severe and complex worldwide, primarily due to shallow underground water tables, distinctive hydrological and geographical characteristics, and groundwater salinization [1]. The Yellow River Delta is a typical coastal saline-alkali region in northern China. Over 60% of this area is affected by varying degrees of salinization [2]. Soil salinity in this region is strongly influenced by precipitation, shallow groundwater, and evaporation, resulting in pronounced seasonal fluctuations [3]. Rainfall promotes salt leaching, whereas dry, high-evaporation periods favor salt accumulation and resalinization in surface soils. Unlike inland saline-alkali soils, which are commonly associated with arid climates and strong evaporative concentration, coastal saline soils in the Yellow River Delta are additionally influenced by marine processes and shallow groundwater dynamics [4]. In coastal saline areas, soil salinity is strongly influenced by seasonal precipitation patterns and elevated groundwater tables. Severe resalinization commonly occurs in natural coastal saline soils during spring [5], and low soil organic matter content and high salinity levels are key factors affecting soil microbial diversity [6]. Therefore, the underlying processes driving these effects remain poorly understood and require further investigation.

Microorganisms play a pivotal role in soil biogeochemistry, including carbon degradation, nitrogen mineralization, nitrogen fixation, and phosphorus solubilization [7]. Moreover, soil microorganisms enhance plant growth and sustain soil health by improving plant stress resistance and promoting overall plant development [8]. It was reported that the contributions of microbial groups, categorized according to their relative abundance in communities, differ substantially with respect to specific ecosystem functions [9,10]. Abundant taxa are microbial groups characterized by high relative abundance across different samples and play a fundamental and indispensable role in nutrient cycling within ecosystems [11,12]. These taxa typically exhibit greater tolerance to environmental stress and possess sufficient biomass and metabolic capacity to maintain high functional performance under stable environmental conditions [13,14]. Changes in the composition and relative abundance of abundant taxa strongly influence overall microbial diversity, whereas the dynamics of rare taxa are critical for explaining microbial community succession, as these taxa may become abundant under stress conditions [13].

Generally, abundant taxa exhibit broader niche breadths and greater environmental adaptability [15]. Under high-stress conditions, microbial niche breadth tends to increase, and alpha diversity declines [16]. Likewise, during ecosystem succession from grassland to forest, niche overlap among microbial nutrient cycling genes decreases, whereas their functional diversity increases [7]. Therefore, under salt stress, the community structure of abundant taxa tends to remain relatively stable. Furthermore, under multiple environmental stressors, soil microorganisms exhibit significantly expanded niche breadth and reduced species diversity [16]. However, the niche characteristics of abundant, moderate, and rare microbial taxa under salt stress remain poorly understood. Consequently, the influence of microbial niche characteristics on community diversity in saline soils warrants further investigation.

Microorganisms can adapt to environmental stress by altering their life-history strategies, which reflect species fitness and associated trade-offs within specific environmental contexts [17]. Taxa with higher ribosomal RNA operon (rrn) copy numbers, often found in Pseudomonadota, Actinomycetota, and Bacteroidota, usually exhibit high reproductive capacity and rapid growth rates [17]. In contrast, taxa with lower rrn copy numbers, more common in Acidobacteriota, Nitrospirota, and Planctomycetota, are characterized by the ability to utilize recalcitrant nutrients and by greater resistance to environmental stress [18]. Environmental disturbances can increase substrate availability, thereby promoting the enrichment of bacteria with higher rrn copy numbers [19]. Conversely, in nutrient-sufficient and non-stressful environments, community assembly is primarily driven by stochastic processes [16]. Environmental stressors such as drought or salinity generally select for microorganisms with higher growth rate, whereas the richness and relative abundance of microorganisms with lower growth rate decline [20]. Collectively, although microbial adaptation to salt stress via shifts in life-history strategies is well recognized, its implications for microbial diversity remain to be further explored.

Coastal saline lands are typically characterized by pronounced salinization during spring, with most existing studies focusing on its impact on soil microbial community composition and crop growth. However, it remains unclear whether salt stress ultimately alters microbial alpha diversity. Field sampling was used to investigate the dynamics of soil properties and microbial communities during natural resalinization in coastal saline soils, with the aim of elucidating the mechanisms driving shifts in microbial diversity. We hypothesize that (1) environmental stress induced by spring salt accumulation in coastal saline soils alters soil microbial community assembly processes, leading to an enhancement in deterministic processes and a decrease in stochastic processes; (2) shifts in microbial community assembly processes influence microbial life-history strategies, leading to an increased relative abundance of microorganisms with higher rrn copy numbers and a decreased relative abundance of microorganisms with lower rrn copy numbers; (3) these shifts in microbial life-history strategies subsequently affect microbial diversity by regulating niche breadth and niche overlap; (4) changes in abundant taxa play a key role in determining the life-history strategies and niche properties of the overall bacterial community. This study aimed to quantify changes in soil salinity and bacterial alpha diversity, identify shifts in community assembly, and examine how life-history strategies and niche properties relate to bacterial diversity, which is important for the biological reclamation of saline soils and the enhancement of soil biological functions.

## 2. Materials and Methods

### 2.1. Site Description

The sampling field characterized by a temperate continental monsoon climate with semi-humid conditions, was located in Wudi County, Shandong Province, China (37°34′ N, 117°52′ E). Average annual air temperature is about 12.6–12.7 °C, and annual potential evaporation reaches roughly 1800 mm. The long-term mean annual precipitation is approximately 575 mm, with nearly 70% of the rainfall concentrated during the summer months from June to August. The depth of the groundwater table at the site ranges between 0.9 and 1.1 m. The soil is classified as a typical loam and exhibits alkaline properties (pH 8.86). According to the IUSS Working Group World Reference Base for Soil Resources, the soil is classified as a Salic Fluvisol [21], corresponding approximately to a Typic Halaquept in USDA Soil Taxonomy [22].

Soil sampling was conducted on 19 March, 15 May, and 14 June, representing the early-spring, late-spring, and early-summer periods, respectively. According to long-term meteorological records from the Wudi meteorological station, mean air temperature increased from approximately 7.5 °C in March to 20.9 °C in May and 25.3 °C in June, while monthly precipitation increased from approximately 7.8 mm to 46.8 and 84.8 mm, respectively. Thus, the three sampling periods encompassed a seasonal transition from relatively cool and dry spring conditions to warmer and increasingly wet early-summer conditions. This seasonal variation in precipitation provides an important climatic context for the alternating leaching and salt accumulation processes characteristic of coastal saline soils.

### 2.2. Soil Sampling and Soil Property Analysis

Each sampling field was arranged in four replicated plots measuring 11 × 13 m. Soil sampling was conducted on 19 March, 15 May, and 14 June from the 0–20 cm soil depth. In each sampling plot, three soil cores were randomly collected in a 1 × 1 m quadrat and combined into a sample. Before sampling the next soil sample, the soil auger was cleared of residual soil and disinfected with 75% ethanol. Soil samples were immediately transferred into sterile sampling bags. Then, soil samples were thoroughly mixed and passed through a 2-mm sieve to remove plant debris, roots, and stones. During sample processing, a separate 2-mm sieve was used for each treatment to minimize cross-contamination, and disposable gloves were replaced. Partial soil samples were immediately stored at −80 °C to preserve DNA integrity prior to molecular analyses. In addition, undisturbed soil cores collected in aluminum boxes (10 cm in diameter) were wet-sieved through 250-μm and 20-μm sieves to separate macroaggregates (>250 μm) and microaggregates (20–250 μm) [23]. Total salt content was tested using the gravimetric method. Soil organic matter (SOM) was determined using potassium dichromate (K_2_Cr_2_O_7_) oxidation with colorimetric detection (organic matter = 1.724 × organic C) [24].

### 2.3. Bacterial 16S rRNA Gene Amplification and High-Throughput Sequencing

The EZNA Soil DNA Kit (Omega Bio-Tek, Inc., Norcross, GA, USA) was used to isolate total genomic DNA from 0.5 g of soil material in accordance with the supplier’s protocol. To characterize bacterial community composition, 336F (GTACTCCTACGGGAGGCAGCA) and 806R (GGACTACHVGGGTWTCTAAT) were used as the universal primers to amplify the hypervariable V3-V4 region of the 16S rRNA gene [25,26]. PCR amplification was performed in triplicate on an ABI GeneAmp 9700 thermal cycler (Applied Biosystems, Foster City, CA, USA). The reaction mixture (total volume 50 μL) contained 5 μL of 10 × Pyrobest Buffer, 4 μL of 2.5 mM dNTPs, 2 μL of 10 μM of each primer, 0.3 μL of Pyrobest DNA Polymerase (2.5 U μL^−1^, TaKaRa Code: DR005A), and 30 ng of template DNA. Sequencing libraries were constructed and sequenced on the Illumina MiSeq platform (Beijing Allwegene Technology Co., Ltd., Beijing, China). The resulting sequence reads were analyzed using QIIME software (v1.17) in combination with the UPARSE workflow. Raw FASTQ datasets were subjected to quality control and filtering procedures implemented in QIIME. High-quality sequences were clustered into operational taxonomic units (OTUs) at 97% sequence similarity using UPARSE, and potential chimeras were detected and eliminated with the UCHIME algorithm. After quality control and chimera removal, a total of 235,199 high-quality sequences were retained across the 12 samples (ranging from 11,857 to 26,854 reads per sample; mean, 19,600), yielding 4799 OTUs. Representative OTU sequences were then taxonomically assigned using the Ribosomal Database Project (RDP) classifier. The raw data have been deposited in the NCBI SRA database under the accession number SRP117226.

### 2.4. Analysis of Abundant, Moderate, Rare Taxa and Different Life-History Strategy Bacteria

Operational taxonomic units (OTUs) were categorized into three broad groups according to their distribution patterns and proportional representation across samples, following previously established frameworks [10]. Specifically, bacterial taxa were subdivided as follows: (i) abundant taxa (AT), including abundances ranged between 0.01% and 1% in all samples and abundances ≥ 1% in some samples; (ii) moderate taxa (MT), characterized by abundances < 0.01% in some samples without ever reaching ≥ 1%; (iii) rare taxa (RT), exhibiting relative abundances < 0.01% across all samples [8,27]. To quantify community-level rrn copy number characteristics associated with high-rrn and low-rrn taxa, the community-weighted mean rRNA operon copy number (CWMrrn) was calculated. The mean 16S rRNA gene operon copy number for each of the six bacterial phyla was retrieved from the rrnDB database (version 5.10) [28,29,30]. Pseudomonadota, Actinomycetota, and Bacteroidota were classified as bacterial phyla with higher mean rrn copy numbers, and Acidobacteriota, Nitrospirota, and Planctomycetota were classified as bacterial phyla with lower mean rrn copy numbers [19,31]. For each sample, CWMrrn was computed as CWMrrn = Σ(rrn_i_ × RA_i_), where rrn_i_ is the phylum-level mean rrn copy number and RA_i_ is the relative abundance of phylum i. Differences in CWMrrn among sampling months were assessed using the Kruskal–Wallis test.

### 2.5. Analysis of Microbial Community

All statistical analyses were conducted in the R environment (version 4.4.3). Bacterial community structural characteristics and composition were analyzed at the phylum level. Prior to diversity analyses, the OTU table was rarefied to an even depth to normalize sequencing depth. Variations in community structure across sampling dates were explored through PCoA (principal coordinate analysis) on the basis of Bray–Curtis dissimilarity index. Bacterial alpha diversity was assessed by calculating the Shannon diversity index. Ecological niche breadth and overlap of bacterial taxa were calculated at the OTU level (97% sequence similarity) using the spaa package in R. Niche breadth was quantified using Levins’ index, which measures the uniformity of each OTU’s relative abundance distribution across samples. Niche overlap between OTU pairs was quantified using Pianka’s index, which ranges from 0 (no overlap) to 1 (complete overlap) [32]. The normalized stochasticity ratio (NST) package in R was used to assess the balance between stochastic and deterministic processes in bacterial community [33].

### 2.6. Statistical Analysis

Differences among measured variables at different stages were tested by one-way analysis of variance (ANOVA). Associations between pairs of variables were examined through Pearson’s correlation coefficients. Significant differences were identified when the *p* value was less than 0.05. The PLS-SEM (partial least squares structural equation modeling) was applied to explore both direct and indirect pathways linking soil salinity, bacterial community assembly processes, bacterial life-history strategies, niche properties, and bacterial alpha diversity. All constructs were specified as reflective and estimated using the centroid weighting scheme (tolerance = 1 × 10^−6^, maximum 100 iterations). The model included seven constructs: soil salinity, NST, high-rrn relative abundance, low-rrn relative abundance, high-rrn niche breadth, low-rrn niche properties, and Shannon diversity. Soil salinity, NST, high-rrn relative abundance, low-rrn relative abundance, high-rrn niche breadth, and Shannon diversity were treated as single-indicator constructs, whereas low-rrn niche properties was modeled as a reflective construct comprising niche breadth and niche overlap. Given the small sample size (N = 12), PLS-SEM was selected for its suitability for small-sample and exploratory analysis. Bootstrapping was performed with 10,000 subsamples using the percentile method, a fixed random seed (12,345), and two-tailed testing at α = 0.05. Path significance was assessed using *t*-values and *p*-values from the inner model, and path stability was assessed using bootstrap standard errors and 95% confidence intervals. Collinearity was evaluated using VIF < 5 considered acceptable. Model fit was assessed using GOF (>0.36) and *R*^2^ for endogenous constructs [34]. All analyses were conducted in R using the plspm package [35].

## 3. Results

### 3.1. Seasonal Dynamics of Soil Physical and Chemical Properties

**Figure 1:**
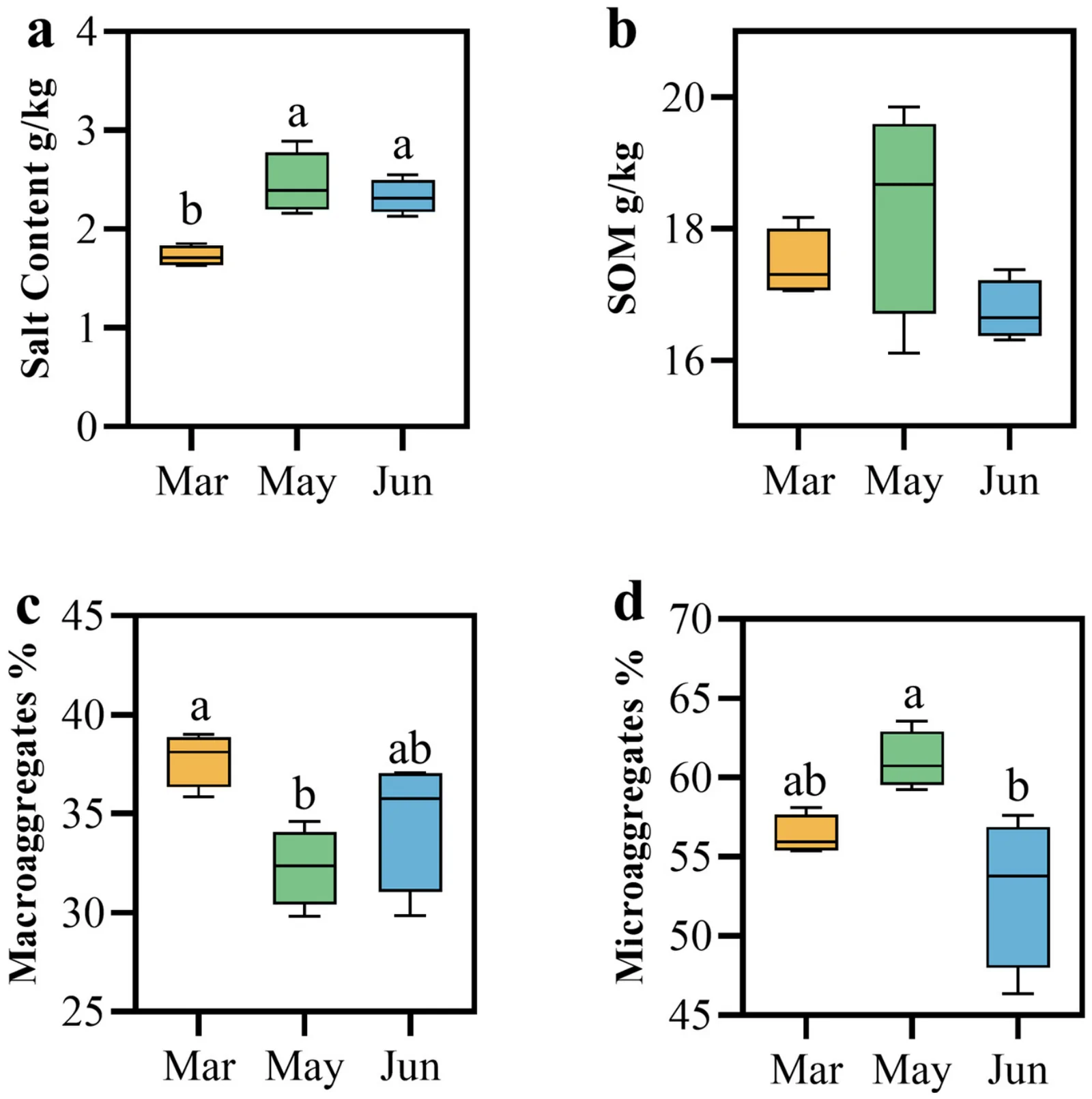
Seasonal dynamics of soil salinity (**a**), soil organic matter (**b**), macroaggregates (**c**) and microaggregates (**d**). The lowercase letters show significant differences between different stages at *p* < 0.05.

Severe resalinization of natural coastal saline soil was observed in May and June, leading to a significant increase in soil salinity during these months (Figure 1a), whereas surface soil organic matter (SOM) did not vary significantly among months (Figure 1b). Additionally, the proportion of soil macroaggregates decreased substantially in May compared with March (Figure 1c), whereas microaggregates increased significantly in May relative to June (Figure 1d).

### 3.2. Seasonal Dynamics of Soil Bacterial Community Composition and Diversity

**Figure 2:**
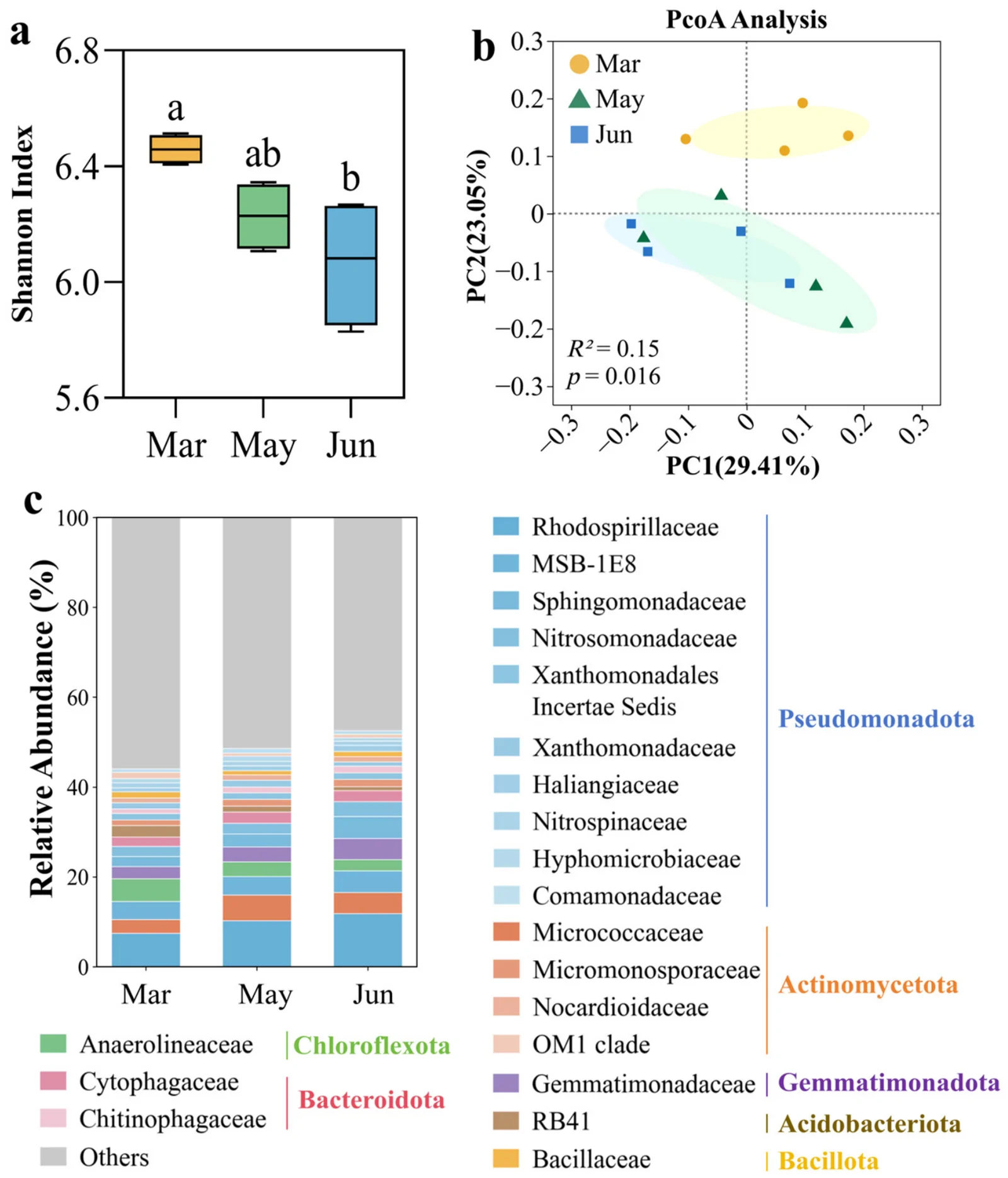
Shannon index illustrating the α diversity of bacterial community (**a**), the lowercase letters show significant differences between different stages at *p* < 0.05. PCoA illustrating the variation in the bacterial community for soil bacteria (**b**), with 95% confidence ellipses shown around the grouped samples based on sampling period, *R*^2^ and *p* values were calculated using ANOSIM (analysis of similarities) with 999 permutations. Stacked bar plot of community composition (**c**), showing the relative abundance of the top 20 bacterial families in different stages. The phylum to which each family belongs is indicated on the right.

The biodiversity index (Shannon) of the soil bacteria decreased steadily and significantly in June compared with March, indicating a pronounced decline in alpha diversity (Figure 2a). PCoA analysis based on OTU data revealed that bacterial community structures clustered distinctly according to low soil salinity (March) and high soil salinity (May and June) (*p* = 0.016, Figure 2b). As shown in Figure 2c, Rhodospirillaceae was the most abundant family (9.87%), followed by Micrococcaceae (4.47%) and MSB-1E8 (4.34%) at family level. From March to June, the relative abundance of Rhodospirillaceae increased from 7.49% to 11.84%, whereas Anaerolineaceae and RB41 decreased from 5.07% to 2.48% and from 2.60% to 0.88%, respectively (Figure 2c). Unidentified families accounted for 23.23% of the total community. At the phylum level, Acidobacteriota, Actinomycetota, Bacteroidota, Chloroflexota, Gemmatimonadota, and Pseudomonadota were the six dominant bacterial phyla across all treatments and sampling stages (Figure S2). The relative abundances of Gemmatimonadota and Pseudomonadota enhanced progressively and significantly from March to June, whereas those of Acidobacteriota, Chloroflexota and Planctomycetota decreased significantly over the same period (Figure S2).

### 3.3. Changes in Bacterial Life-History Strategy and Niche Properties of Different Taxa

**Figure 3:**
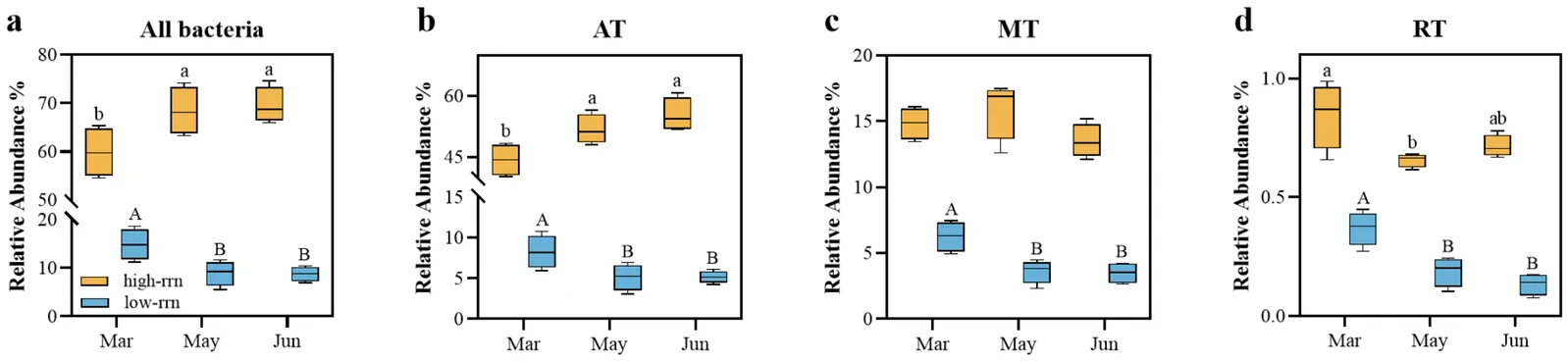
Bacterial relative abundance of (**a**) all taxa (all), (**b**) abundant taxa (AT), (**c**) moderate taxa (MT) and (**d**) rare taxa (RT) with different life-history strategies (high-rrn and low-rrn). The lowercase letters show significant differences in high-rrn bacteria between different stages at *p* < 0.05, the uppercase letters show significant differences in low-rrn bacteria between different stages at *p* < 0.05. High-rrn: Pseudomonadota, Actinomycetota, and Bacteroidota; low-rrn: Acidobacteriota, Nitrospirota, and Planctomycetota.

The bacterial phyla with higher growth rate exhibited higher rrn copy numbers than those with lower growth rate. Pseudomonadota (5.82), Actinomycetota (3.79), and Bacteroidota (4.18) were described as phyla with higher rrn copy numbers (high-rrn), whereas Acidobacteriota (1.34), Nitrospirota (1.40), and Planctomycetota (2.04) were described as phyla with lower rrn copy numbers (low-rrn) (Figure S1a). The community-weighted mean rrn (CWMrrn) increased significantly from March to June (Figure S1b). Compared with March, the proportions of all and abundant high-rrn bacteria increased significantly in May and June (Figure 3a,b), whereas the relative abundance of rare high-rrn bacteria declined significantly in May (Figure 3d). The changes in phyla with lower rrn copy numbers across different taxa were consistent with those observed for all phyla with lower rrn copy numbers, showing significant declines in May and June relative to March (Figure 3a–d).

**Figure 4:**
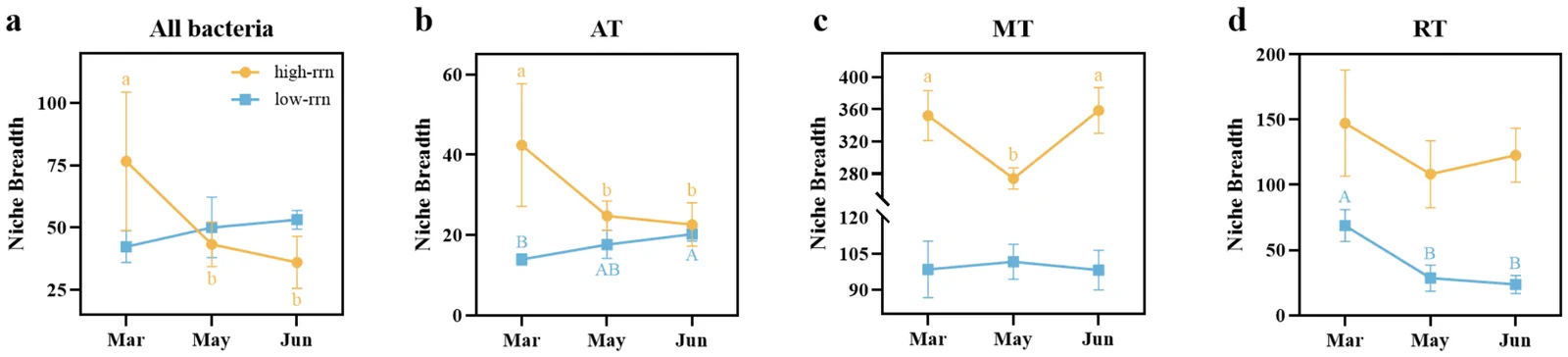
Bacterial niche breadth of (**a**) all taxa (all), (**b**) abundant taxa (AT), (**c**) moderate taxa (MT) and (**d**) rare taxa (RT) with different life-history strategies (high-rrn and low-rrn). The lowercase letters show significant differences in high-rrn bacteria between different stages at *p* < 0.05, the uppercase letters show significant differences in low-rrn bacteria between different stages at *p* < 0.05. High-rrn: Pseudomonadota, Actinomycetota, and Bacteroidota; low-rrn: Acidobacteriota, Nitrospirota, and Planctomycetota.

The niche breadths of all and abundant phyla with higher rrn copy numbers decreased significantly in May and June compared with March (Figure 4a,b), whereas those of abundant phyla with lower rrn copy numbers increased significantly in June (Figure 4b). Additionally, the niche breadth of moderate high-rrn bacteria declined significantly in May (Figure 4c), and that of rare low-rrn bacteria also declined significantly in May and June relative to March (Figure 4d).

**Figure 5:**
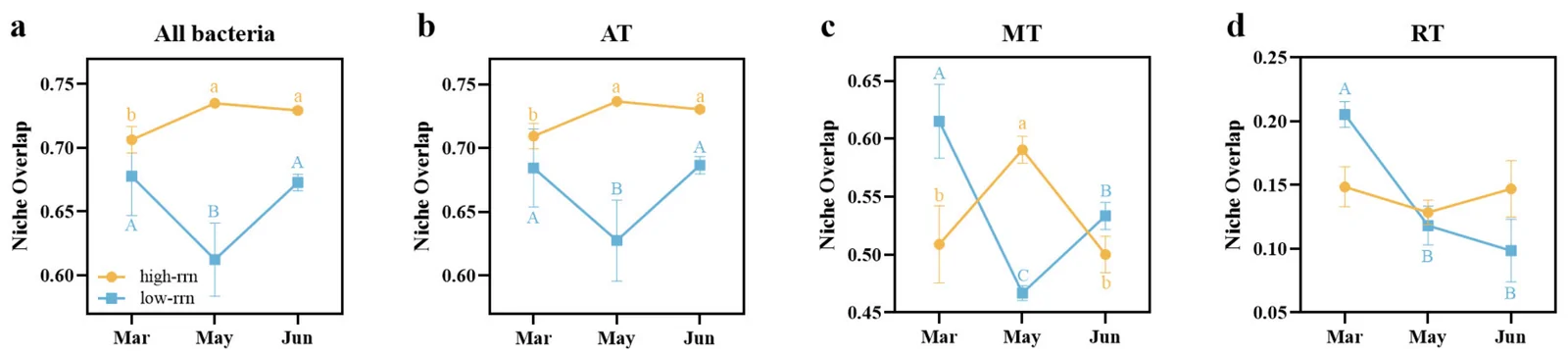
Bacterial niche overlap of (**a**) all taxa (all), (**b**) abundant taxa (AT), (**c**) moderate taxa (MT) and (**d**) rare taxa (RT) with different life-history strategies (high-rrn and low-rrn). The lowercase letters show significant differences in high-rrn bacteria between different stages at *p* < 0.05, the uppercase letters show significant differences in low-rrn bacteria between different stages at *p* < 0.05. High-rrn: Pseudomonadota, Actinomycetota, and Bacteroidota; low-rrn: Acidobacteriota, Nitrospirota, and Planctomycetota.

The niche overlap of all and abundant phyla with higher rrn copy numbers increased substantially in May and June, whereas that of phyla with lower rrn copy numbers declined markedly in May relative to other stages (Figure 5a,b). Within the moderate taxa, niche overlap of high-rrn bacteria reached its highest level in May, whereas that of low-rrn bacteria reached its lowest level during the same period (Figure 5c). Changes in niche overlap among rare high-rrn bacteria were not substantial, whereas that of low-rrn bacteria decreased significantly in May and June relative to March (Figure 5d).

### 3.4. Changes in Community Assembly Mechanisms of Different Strategy Bacteria

**Figure 6:**
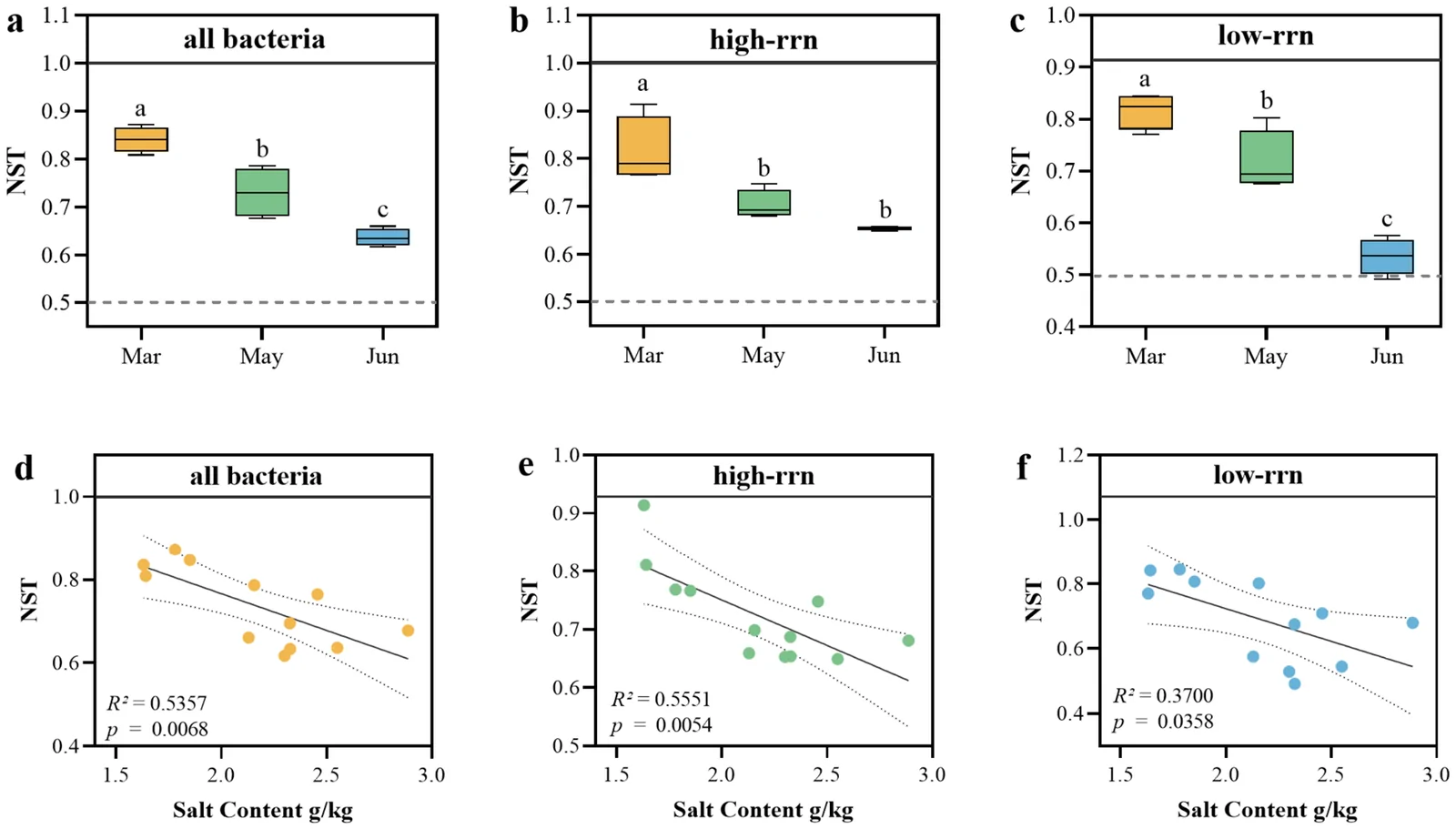
Normalized stochasticity ratio (NST) and correlation between NST and salinity. NST of all bacteria (**a**), high-rrn bacteria (**b**) and low-rrn bacteria (**c**). Pearson correlation between NST and salinity of all bacteria (**d**), high-rrn bacteria (**e**) and low-rrn bacteria (**f**). The lowercase letters show significant differences between different stages at *p* < 0.05.

The NST (normalized stochasticity ratio) values for all bacteria, phyla with higher rrn copy numbers and phyla with lower rrn copy numbers decreased progressively and substantially from March to June (Figure 6a,c). Correlation analyses between soil salt content and NST across different life-history strategies revealed significant negative relationships (*p* < 0.05), indicating that increasing soil salinity suppresses stochastic processes while promoting deterministic processes (Figure 6d–f).

### 3.5. Changes in Alpha Diversity and Dominant Function of Abundant Taxa

**Figure 7:**
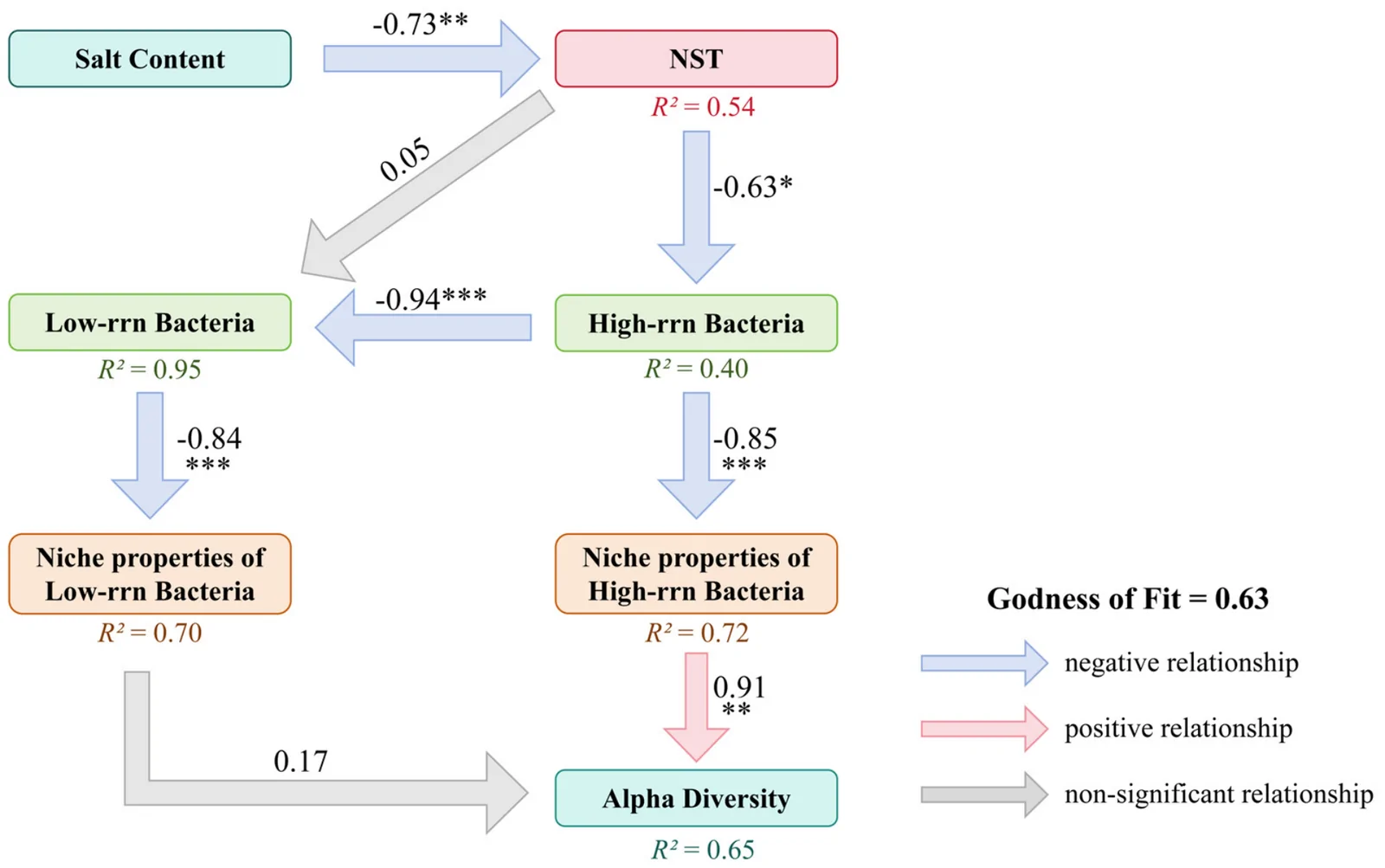
Partial least squares structural equation model (PLS-SEM) of soil salt content, community assembly mechanisms (NST), the relative abundance of high and low-rrn bacteria, the niche properties of high and low-rrn bacteria, and bacterial alpha diversity. Red arrows indicate significant positive relationships, blue arrows indicate significant negative relationships, and gray arrows indicate non-significant relationships. Numbers on the arrows are standardized path coefficients, with asterisks denoting significance levels (* *p* < 0.05, ** *p* < 0.01, *** *p* < 0.001). *R*^2^ values represent the proportion of variance explained for each endogenous variable.

The PLS-SEM results showed acceptable fit, with a goodness-of-fit (GOF) value of 0.63, which was well above the recommended cutoff of 0.36 [36]. The *R*^2^ values of the endogenous constructs ranged from 0.40 to 0.95, with an *R*^2^ of 0.65 for bacterial alpha diversity. Soil salinity was significantly associated with bacterial alpha diversity (*p* < 0.05; Figure 7). Specifically, increased soil salinity was negatively associated with the normalized stochasticity ratio (NST) (*p* < 0.01), and lower NST was in turn associated with higher relative abundance of high-rrn bacterial taxa (*p* < 0.05). The relative abundance of bacterial phyla with higher rrn copy numbers was negatively associated with their niche properties (*p* < 0.01), which were in turn positively associated with bacterial alpha diversity (*p* < 0.05; Figure 7). In contrast, the paths involving low-rrn taxa were generally weaker. The path from NST to low-rrn relative abundance was not significant (*p* = 0.649), and the path from low-rrn niche properties to Shannon diversity was not significant (*p* = 0.512). The full path coefficients, significance tests, and bootstrap 95% confidence intervals are provided in Table S1.

A heatmap was used to display the relative abundance and niche properties of all bacteria with higher and lower rrn copy numbers across abundant, moderate, and rare taxa (Figure S3). Statistical analysis revealed that abundant taxa exhibited significant positive relationships with the relative abundance and niche traits of all bacteria across diverse life-history strategies (*p* < 0.001). In contrast, moderate and rare taxa representing different life-history strategies did not show substantial correlations with all bacteria in terms of relative abundance or niche properties, indicating that abundant taxa are the dominant drivers shaping life-history strategy composition, niche breadth and niche overlap (Figure S3).

## 4. Discussion

### 4.1. Soil Salinization Increases the Contribution of Deterministic Processes in Soil Bacterial Community

Increased soil salinity is a defining characteristic of the spring resalinization in coastal saline lands [36]. This natural process provides an ideal setting for investigating microbial community assembly mechanisms under environmental disturbances. In this study, we found that the bacterial community assembly shifted from stochastic to deterministic processes in response to resalinization (Figure 1a, Figure 6 and Figure 7). This pattern may be attributed to soil salinity acting as a strong environmental stressor that directly influences bacterial community assembly [37,38]. Consequently, we propose that salinization in coastal saline soils is a direct and crucial factor influencing deterministic assembly processes in soil bacterial community.

Salt stress diminishes the roles of ecological drift and random dispersal in microbial community assembly while reinforcing environmental filtering [39]. Niche theory further supports these findings, suggesting that under environmental stress, bacterial stress tolerance becomes a key determinant of survival [40]. At the family level, the bacterial community was dominated by Rhodospirillaceae, Micrococcaceae, Anaerolineaceae, and Gemmatimonadaceae (Figure 2c), which was consistent with other coastal saline-alkali soil studies [41]. At the phylum level, this composition is broadly consistent with other saline soils, where Pseudomonadota and Actinomycetota often dominate [42]. This finding provides microbial-scale evidence supporting the stress gradient hypothesis, which posits that increasing abiotic stress alters community assembly mechanisms. Moreover, this shift in assembly mechanisms occurred over a short period during spring resalinization (March to June) in coastal saline lands, indicating that bacterial communities respond to rapid environmental changes not only through species replacement but also through modifications of their assembly processes, with an increase in the influence of deterministic processes.

### 4.2. Deterministic Processes Shift Bacterial Life-History Strategies

Salt stress induced by spring resalinization in coastal saline lands alters bacterial community assembly mechanisms by strengthening environmental filtering, while bacterial life-history strategies concurrently respond to changes in soil salinity [43]. We found that as soil salt stress intensifies and deterministic processes become more dominant, the bacterial community shifted from low-rrn bacteria toward high-rrn bacteria (Figure 3a). This pattern was consistent with theoretical hypothesis that shifts in microbial community assembly mechanisms drive corresponding changes in microbial life-history strategies. Moreover, soil organic matter content did not vary significantly across the three sampling periods in this study (Figure 1b). This finding suggests that salinity acts as a key environmental driver independent of changes in resource availability.

PLS-SEM further revealed that the strengthening of deterministic processes in bacterial community assembly represents a key pathway through which environmental stress alters bacterial life-history strategies (Figure 7). As deterministic processes strengthened, high-rrn taxa were positively selected, which were characterized by rapid responses to environmental changes and allocating greater energy to reproduction and regulation [44,45]. Conversely, low-rrn bacteria thrive in stable and competitive environments through efficient resource utilization, and experience a reduction in competitive advantage under conditions where stochastic processes are suppressed, leading to a decline in their relative abundance [16,46]. Collectively, these results suggest that under salt stress, the increased role of deterministic processes in bacterial community assembly is associated with changes in bacterial community life-history strategies and with a shift from bacteria with lower rrn copy numbers toward those with higher rrn copy numbers.

### 4.3. Shifts in Bacterial Life-History Strategies Affect Alpha Diversity by Regulating Niche Properties

Our results showed that bacterial life-history strategies shifted from low-rrn bacteria to high-rrn bacteria, and that the niche breadth and niche overlap of both life-strategy bacteria were significantly altered under salt stress (Figure 3a, Figure 4a and Figure 5a). These results support the theoretical hypothesis that shifts in bacterial life-history strategies influence community structure by regulating niche characteristics [16]. Under conditions without salt stress, low-rrn bacteria could exploit a wider variety of resources due to their greater resource utilization capacity. This increased their niche breadth and maintained lower niche overlap through niche differentiation, thereby facilitating species coexistence [46]. As intensified salt stress enhances deterministic processes, strong environmental filtering increasingly selects for high-rrn bacteria [19]. To cope with osmotic stress, these taxa allocate much of their metabolic resources to tolerance mechanisms and the use of readily degradable organic matter, which was a strategy linked to simplified and convergent resource use. Concurrently, directional selection toward taxa with similar physiological bases for salt tolerance is accompanied by a significant reduction in niche breadth [16,47].

Furthermore, bacterial alpha diversity decreased significantly under salt stress conditions (Figure 2a). From the perspective of niche theory, species coexistence and the maintenance of diversity depend on sufficient niche differentiation to alleviate competitive exclusion [47]. In this study, although soil organic matter content did not vary significantly across sampling periods, the reduced niche breadth of high-rrn bacteria indicates a narrowing of the resource spectrum exploited by these organisms. This suggests that bacteria with higher rrn copy numbers do not fully exploit available environmental resources, thereby limiting the species carrying capacity of the community [48]. Additionally, the significant increase in niche overlap among these taxa may further constrain the range of resources exploited by bacteria with higher rrn copy numbers, consequently decreasing their diversity. Simultaneously, the decline in the relative abundance and community contribution of bacteria with lower rrn copy numbers, which typically exhibit wider niche breadth and low overlap, further diminishes the overall niche differentiation within the community [16]. Results from the PLS-SEM further demonstrate that shifts in bacterial life-history strategies ultimately reduce bacterial alpha diversity by influencing niche breadth (Figure 7).

### 4.4. Linkages Between Abundant Taxa and Community-Level Life-History Strategies and Niche Properties

In this study, the relative abundances of two life-history strategy bacteria within abundant taxa are significantly positively correlated with those of the overall microbial community (Figure S3). This indicates that abundant taxa may contribute to community-level patterns [9]. However, because abundant taxa are a subset of the overall community, this relationship is partly mathematically inherent. Furthermore, the niche breadth and niche overlap indices of high and low-rrn abundant bacteria taxa are positively correlated with the relative abundances of corresponding strategist groups in the overall community (Figure S3). This demonstrates that abundant taxa exert an impact on the niche properties of the entire microbial community. Therefore, abundant taxa occupy a leading position in resource acquisition, environmental adaptation, and population expansion, thereby driving the entire community toward similar life-history strategies [13].

**Figure 8:**
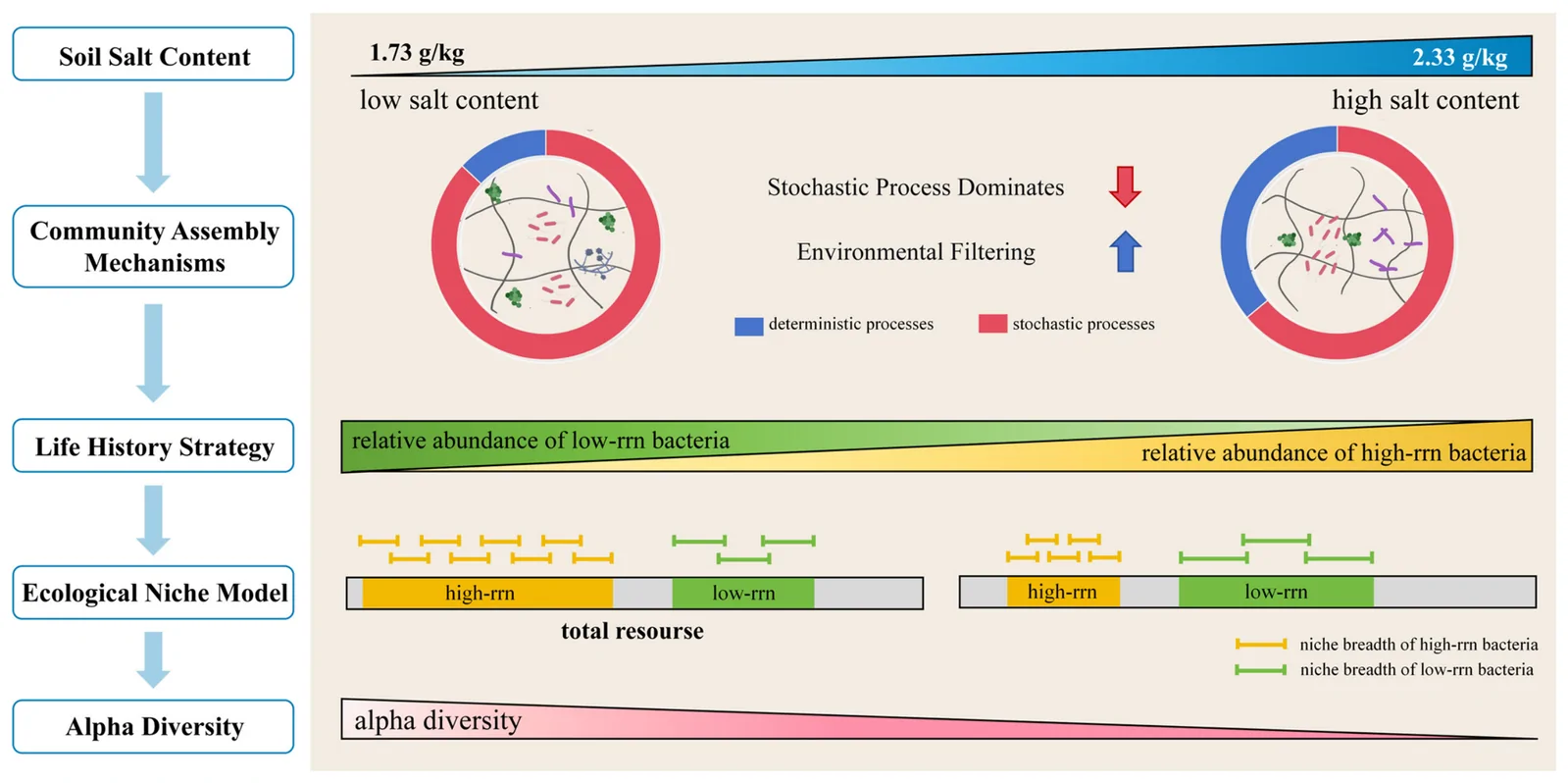
A conceptual overview of soil salinity affects soil bacterial alpha diversity by mediating community assembly mechanisms, bacterial life-history strategy and their niche breadth and niche overlap.

In summary, abundant taxa appear to be associated with the overall life-history strategies, niche breadth, and niche properties of soil microbial communities (Figure 8). This result suggests that, along the pathway from environmental changes to bacterial alpha diversity, changes in abundant taxa are associated with shifts in the life-history strategies and niche characteristics of the overall bacterial community, partly reflecting the mathematical dependence of community-level metrics on abundant taxa. Specifically, spring resalinization in coastal saline lands increases soil salinity and enhances deterministic processes of soil bacteria. This shift coincides with a change in the life history strategies from phyla with lower rrn copy numbers to phyla with higher rrn copy numbers. This pattern is accompanied by altered niche properties of abundant taxa and lower alpha diversity.

## 5. Conclusions

In this single-year study conducted in coastal saline soils of Shandong, China, spring resalinization was associated with reduced bacterial α-diversity and with changes in bacterial community composition, community assembly processes, and life-history strategy properties. From March to June, soil salt content increased significantly, accompanied by a shift toward stronger deterministic processes, a change in the life-history strategies of abundant taxa from bacterial phyla with lower rrn copy numbers to bacterial phyla with higher rrn copy numbers, and altered niche properties. These results suggest that resalinization was associated with changes in bacterial diversity through multiple ecological processes, including shifts in community assembly, life-history strategies, and niche differentiation. However, because these findings are based on one sampling year and one region, the generality of this pathway across years and sites remains to be tested. These findings provide a basis for developing biologically based remediation strategies for coastal saline soils.

## Data Availability

The data presented in this study are openly available in NCBI at https://www.ncbi.nlm.nih.gov/sra/?term=SRP117226 (accessed on 7 October 2026), reference number SRP117226.

## Supplementary Materials

The following supporting information can be downloaded at: https://www.mdpi.com/article/10.3390/biology15201816/s1, Figure S1: Ribosomal RNA operon (rrn) copy number of the six bacterial phyla (a); community-weighted mean ribosomal RNA operon copy number (CWMrrn) of the bacterial community across sampling periods (b). The lowercase letters show significant differences between different stages at *p* < 0.05, Figure S2: Stacked bar plot of community composition shows the relative abundance of soil top twelve phyla bacteria communities in different stages, Figure S3: The heatmap displays Pearson correlation coefficients between relative abundance, niche breadth and niche overlap of different bacterial taxa with different life-history strategies. Values in cells represent correlation coefficients. Colors denote the strength and direction of correlations: red indicates positive correlations, blue indicates negative correlations, and color intensity corresponds to the magnitude of the correlation coefficient, Table S1: Path coefficients, significance tests, and bootstrap 95% confidence intervals of the PLS-SEM model.
