# Supplementary material for: Resalinization Is Associated with Bacterial Alpha Diversity by Influencing Life-History Strategies, and Niche Properties in Coastal Saline Soils
Source: Biology (Basel). 2026 Oct 12;15(20):1816. doi: 10.3390/biology15201816 (PMC13701363; doi:10.3390/biology15201816)
Supplement: Supplementary file 1 [file biology-15-01816-s001.zip › biology-4542375-supplementary.pdf]

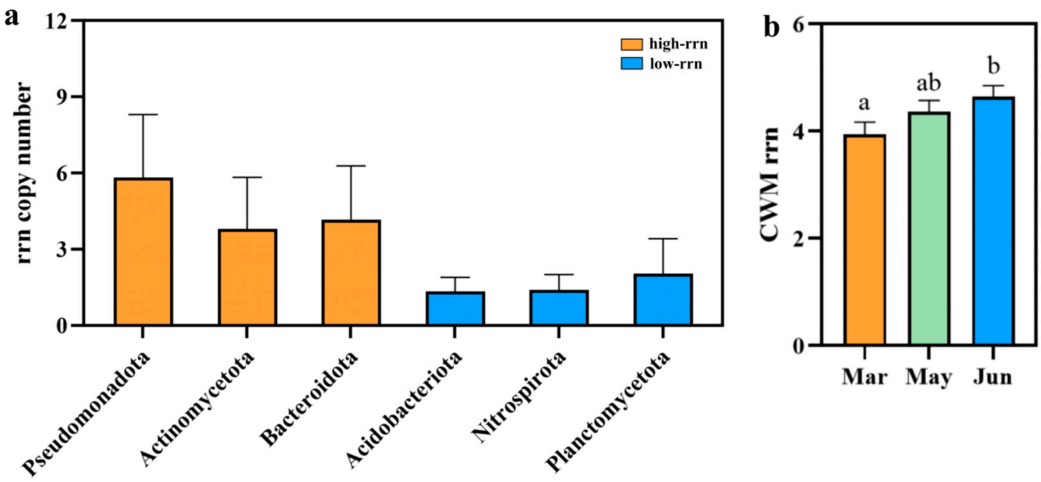

Figure S1 Ribosomal RNA operon (rrn) copy number of the six bacterial phyla (a); community-weighted mean ribosomal RNA operon copy number (CWMrrn) of the bacterial community across sampling periods (b). The lowercase letters show significant differences between different stages at $p < 0.05$.

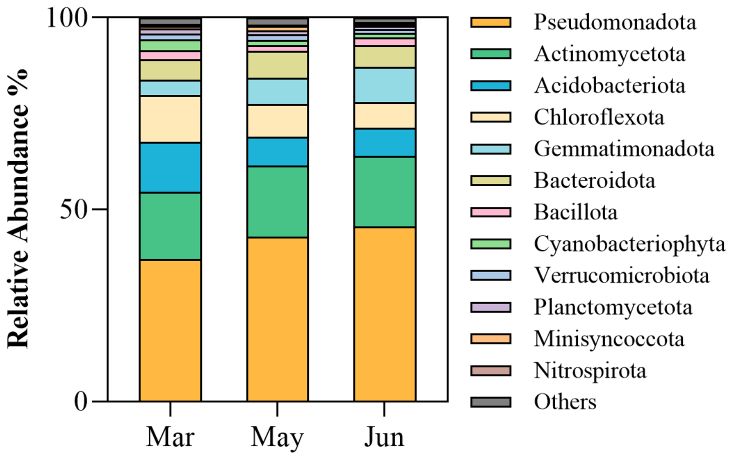

Figure S2 Stacked bar plot of community composition shows the relative abundance of soil top twelve phyla bacteria communities in different stages.

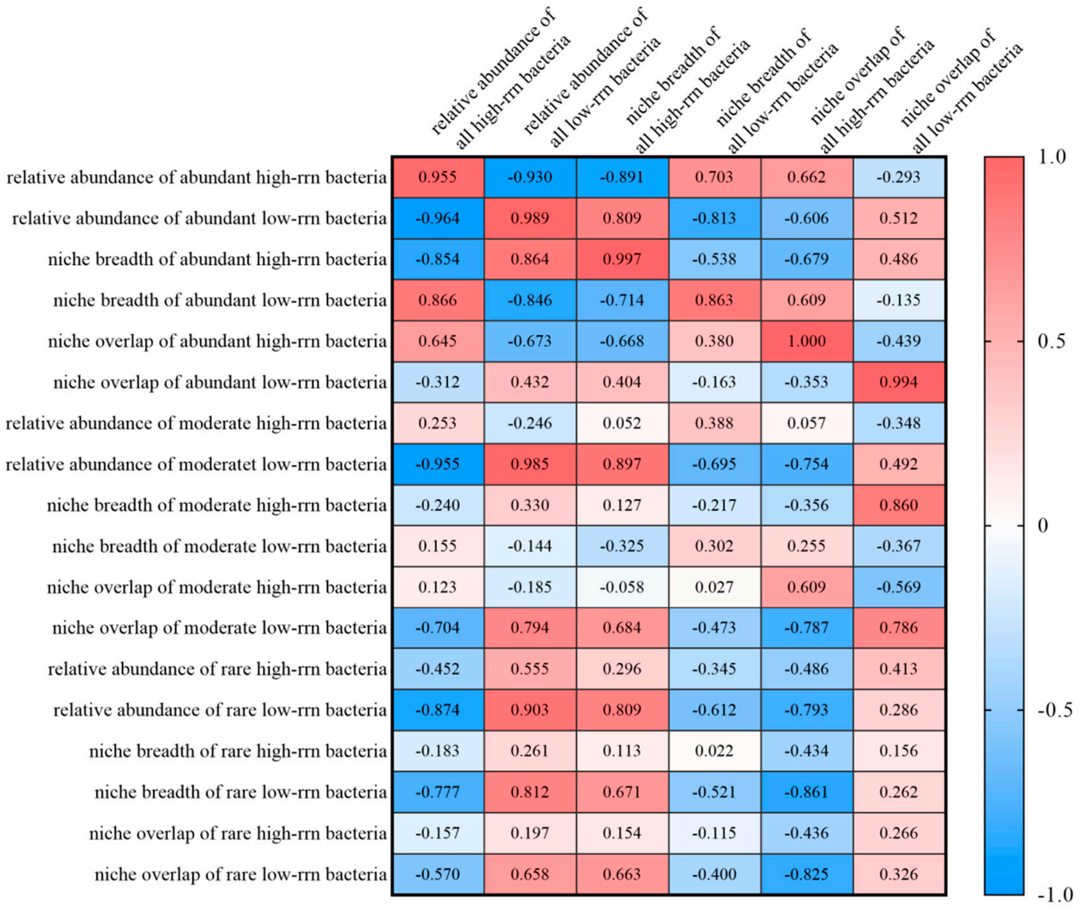

Figure S3 The heatmap displays Pearson correlation coefficients between relative abundance, niche breadth and niche overlap of different bacterial taxa with different life-history strategies. Values in cells represent correlation coefficients. Colors denote the strength and direction of correlations: red indicates positive correlations, blue indicates negative correlations, and color intensity corresponds to the magnitude of the correlation coefficient.

Table S1 Path coefficients, significance tests, and bootstrap 95% confidence intervals of the PLS-SEM model.

Path	Path coefficient	Bootstrap SE	Bootstrap 95% CI	*t*-value	*p*-value
Soil salinity → NST	-0.732	0.115	[-0.905, -0.466]	-3.40	0.007 **
NST → high-rrn relative abundance	-0.635	0.151	[-0.875, -0.303]	-2.60	0.027 *
NST → low-rrn relative abundance	0.047	0.126	[-0.099, 0.415]	0.47	0.649
High-rrn relative abundance → low-rrn relative abundance	-0.942	0.108	[-1.042, -0.613]	-9.39	<0.001 ***
High-rrn relative abundance → high-rrn niche properties	-0.847	0.143	[-0.960, -0.397]	-5.04	<0.001 ***
Low-rrn relative abundance → low-rrn niche properties	0.838	0.388	[-0.667, 0.956]	4.85	<0.001 ***
High-rrn niche properties → Shannon diversity	0.910	0.217	[0.324, 1.284]	3.61	0.006 **
Low-rrn niche properties → Shannon diversity	-0.172	0.285	[-0.606, 0.645]	-0.68	0.512

The *t*-values and *p*-values are from the inner model, whereas bootstrap standard errors (SE) and 95% percentile confidence intervals (CI) are based on 10,000 subsamples with a fixed random seed of 12345. Given the small sample size (N = 12), some paths showed skewed bootstrap distributions; for these paths, the percentile 95% CI crossed zero even though the inner-model *p*-value was significant. Such paths should be interpreted with caution as statistically unstable. * $p < 0.05$; ** $p < 0.01$; *** $p < 0.001$.
